# Suspected Outbreak of Riboflavin Deficiency among Populations Reliant on Food Assistance: A Case Study of Drought-Stricken Karamoja, Uganda, 2009–2010

**DOI:** 10.1371/journal.pone.0062976

**Published:** 2013-05-02

**Authors:** Erin K. Nichols, Leisel E. Talley, Nelly Birungi, Amanda McClelland, Elizabeth Madraa, Agnes B. Chandia, Jacqueline Nivet, Rafael Flores-Ayala, Mary K. Serdula

**Affiliations:** 1 Epidemic Intelligence Service assigned to the Division of Nutrition, Physical Activity, and Obesity, Centers for Disease Control and Prevention, U.S. Public Health Service Commissioned Corps, Atlanta, Georgia, United States of America; 2 Centers for Disease Control and Prevention/International Emergency and Refugee Health, Atlanta, Georgia, United States of America; 3 UNICEF-Uganda Country Office, Kampala, Uganda; 4 Concern Worldwide/Emergency Response Team, Kampala, Uganda; 5 Ministry of Health of Uganda, Nutrition Unit, Kampala, Uganda; 6 United Nations World Food Programme, Office of the Country Director, Kampala, Uganda; 7 Centers for Disease Control and Prevention/Division of Nutrition, Physical Activity, and Obesity, International Micronutrient Malnutrition Prevention and Control Team, Atlanta, Georgia, United States of America; The George Washington University Medical Center, United States of America

## Abstract

**Background:**

In 2009, a humanitarian response was launched to address a food security and livelihoods crisis in Karamoja, Uganda. During a polio immunization campaign in mid-August 2009, health workers in Nakapiripit District reported a concern about an increase in mouth sores, or angular stomatitis (AS) and gum ulcerations, among children in one village, and an investigation was launched.

**Objective:**

This article describes the investigation, lessons learned, and provides guidance for monitoring micronutrient deficiencies among populations receiving food assistance.

**Design:**

An investigation into a suspected outbreak of riboflavin (vitamin B2) deficiency was initiated, including a rapid assessment, mass screening, a convenience sample collection of blood specimens (n = 58 symptomatic cases and n = 18 asymptomatic individuals), and analysis of the general food ration (70% ration).

**Results:**

Findings showed signs of AS in only 399 (0.2%) of 179,172 screened individuals, including adults and children. Biochemical analysis confirmed riboflavin deficiency in 84.5% of specimens from symptomatic individuals and 94.4% of specimens from asymptomatic individuals. Ration distribution data showed that 55% of distributions provided less than half the riboflavin RDA.

**Conclusion:**

Evidence was insufficient to confirm an actual outbreak of riboflavin deficiency, though the present investigation adds further documentation that micronutrient deficiencies continue to persist among populations in emergency settings. This article describes challenges, lessons learned, and guidance for monitoring micronutrient deficiencies among food assistance recipients, including: ongoing nutrition monitoring and surveillance; training and sensitization about micronutrient deficiencies, sensitization of the population about locally-available food, and identifying ways to improve micronutrient interventions.

## Introduction

The Karamoja Region of Uganda is a semi-arid area where the majority of the population subsists through agro-pastoral or pastoral livelihoods. In 2009 an acute food security and livelihoods crisis with widespread reliance on food assistance and 10.9% global acute malnutrition (GAM) resulted from three years of successive climatic shocks, extensive crop failure, and disease that decimated both the region’s livestock and crops [1]. A humanitarian response was launched and included a general food distribution. A Comprehensive Food Security Vulnerability Analysis published by World Food Programme (WFP) in 2009 determined that the general population was able to access approximately 30% of their food and nutrient requirements on their own [2]. Thus the planned general ration for the Karamoja Region between April and December 2009 was designed to meet 70% of the daily energy requirement, 1,470 of the recommended 2,100 Kilocalories per person per day. The planned ration included maize grain, dried beans, vegetable oil, corn soy blend, and iodised salt.

During an immunization campaign in mid-August 2009, health workers in Karamoja (Lorengedwat Subcounty, Nakapiripirit District), reported a concern about an increase in mouth sores and gum ulcerations among children in one village. Health staff visited surrounding villages to assess the extent of the problem and identified a number of additional cases. A diagnosis of angular stomatitis (AS) was posited. AS is characterized by bilateral thinning or fissuring of the mouth angles, cheliosis, and glossitis. While AS can be caused by a variety of factors, including overclosure of the mouth (as in people with no teeth), excessive drooling, anemia, and viral syndromes [3], [4], it is most commonly attributed to riboflavin deficiency, of which the functional consequences include a decrease in iron absorption and utilization [5] and attention span and motor skills deficits [6]. Few formal investigations of AS and riboflavin deficiency have been conducted [7].

**Figure 1:**
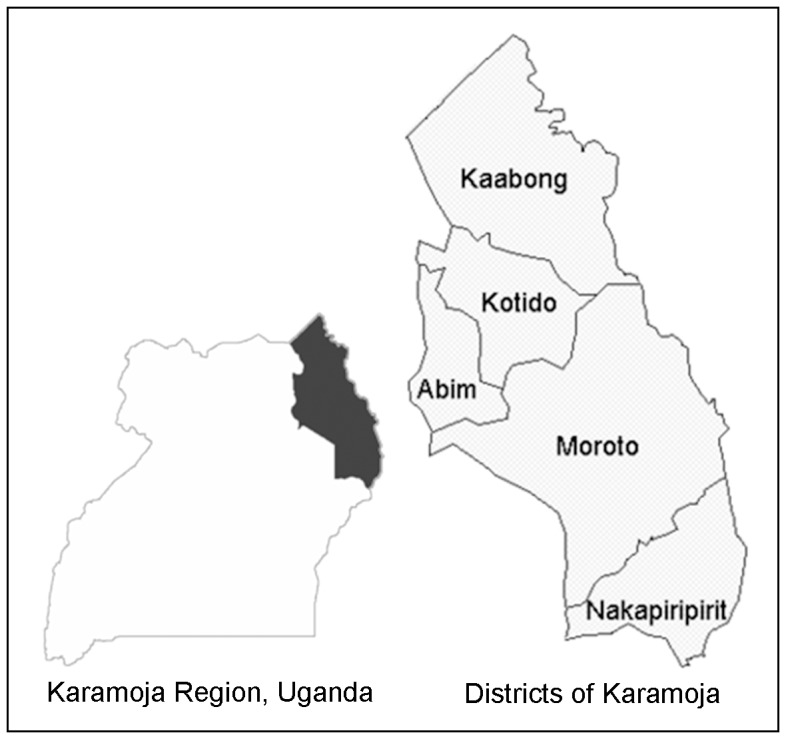
Map of the Karamoja Region and Districts, Uganda.

In response to the suspected outbreak, health officials and UNICEF initiated an investigation in Karamoja to determine the extent of AS and risk factors for possible riboflavin deficiency. This article focuses on activities in Nakapiripirit District (see **Figure 1**) and describes the investigation, lessons learned, and guidance for monitoring micronutrient deficiencies among populations receiving food assistance.

## Methods and Results

The investigation occurred between September 2009 and February 2010. Multiple methods were used in the investigation, including a rapid assessment in Lorengedwat Subcounty (September 2009), a mass screening throughout Nakapiripirit District (end September-November 2009), a convenience sample collection of blood specimens in two subcounties of the District (November 2009), and food ration analysis for a district adjacent to Nakapiripirit District (January-February 2010).

### Ethics Statement

The investigation was designated as public health practice (i.e. non-research) by the CDC Institutional Review Board and thus did not undergo Human Subjects Review.

### Rapid Assessment

On September 4, 2009, health workers conducted a rapid assessment in Lorengedwat Subcounty to identify cases of AS and assess potential associations with other factors including diet. A team comprised of local aid agency and government partners visited health facilities in five villages and observed 130 cases with active mouth sores or gum ulcerations among all people at the health facilities visited. The team informally spoke to a convenience sample of Lorengedwat residents (men, women youth, elderly, opinion leaders) and learned that households were experiencing diminished food access and availability. Residents reported subsisting on maize mash and beans during the week prior to the screening day, and children and adults commonly consumed residue from locally brewed maize or sorghum beer (*kwete).* Local seasonal foods, such as wild leaves and pumpkins, were unavailable due to the drought. Furthermore, some households reported limited access to food due to disruptions in the distribution of the WFP general rations. While milk access had declined during recent years, no abrupt reduction occurred prior to the appearance of AS.

Within two weeks of the rapid assessment, UNICEF procured a stock of vitamin B complex and micronutrient tablets for treatment and prevention of the suspected riboflavin deficiency. Considering the lack of diversity of the diet and limited micronutrient-rich foods consumed by people in Lorengedwat Subcounty, health staff distributed to children and adults a preventive dose of INTAPLEX vitamin B complex tablets (Regal Pharmaceuticals Ltd., Nairobi, Kenya; including 1 mg vitamin B_1,_ 1 mg vitamin B_2,_ and 15 mg vitamin B_3_ per tablet; two tablets per day for 30 days). To pregnant and lactating women in the community, staff distributed micronutrient tablets (UNICEF Copenhagen; including 1.4 mg vitamin B_1_, 1.4 mg vitamin B_2_, 1.4 mg vitamin B_2_, 18 mg vitamin B_3_, 1.9 mg vitamin B_6_, 2.6 µg vitamin B_12_, 800 RE retinol, 10 mg vitamin E, 200 I.U. vitamin D, 400 µg folic acid, 70 mg vitamin C, 30 mg iron fumarate or iron sulphate, 15 mg zinc sulphate, 2 mg copper, 65 µg selenium, and 150 µg iodine per tablet; one tablet per day for 30 days).

### Mass Screening

To determine the extent of the problem, after distribution of vitamin B complex and micronutrient tablets, staff and Village Health Teams (VHTs) from the Lorengedwat Health Center (operating at the subcounty level) conducted a house-to-house mass screening (including all individuals) for AS cases in September/October 2009. Trained by UNICEF and Concern Worldwide, screeners identified cases using photo cards generated from rapid assessment findings; cases were defined as individuals with sores in the corners of the mouth, lips, and/or gums. In addition to recording signs of AS, the team also documented sex, age, subcounty, and pregnancy and/or lactation status (when applicable) for all screened individuals. Based on the initial findings from Lorengedwat Subcounty, mass screening activities were expanded to the remaining subcounties of Nakapiripirit District during house-to-house polio campaign activities in October/November 2009. The subsequent screening activities were implemented by the district health staff and VHTs. Health facilities were provided with the photo cards to assist with case identification.

In Lorengedwat Subcounty, workers screened 4,579 children and adults for AS in September/October 2009, 51% of the projected population by the Ugandan Bureau of Statistics. In the rest of Nakapiripirit District (exclusive of Lorengedwat Subcounty), health workers screened an estimated 80% of the population (174,593 persons). Screeners identified 110 AS cases (2.4%) in Lorengedwat Subcounty and 289 cases (0.2%) in the rest of Nakapiripirit District for a total of 399 (0.2%) AS cases in the District (not including those identified during the rapid assessment). Nearly half of cases (49.6%) occurred in individuals between 0 and 9 years of age. Approximately half of cases were male (**Table 1**).

**Table 1:** Demographics of angular stomatitis cases detected during mass screenings in Nakapiripirit District – Uganda, September – November, 2009.

Demographic	Number of Cases	Percentage of Cases
TOTAL	399	100.0%
Age (years)		
0 to 9	198	49.6%
10 to 19	67	17.0%
20+	134	33.4%
Sex		
Male	201	50.4%
Female	198	49.6%

Following UNICEF guidelines, workers treated positive cases with a therapeutic dose of vitamin B complex (content as previously described; six months to ten years of age: seven tablets per day divided into two doses for four days; over ten years of age: 15 tablets per day divided into two doses for four days). Workers instructed persons identified as cases to return after four days for follow-up and to receive a prophylactic dose of vitamin B complex (content as previously described; children and non-pregnant/non-lactating adults: two tablets per day for 30 days) or micronutrient tablets (content as previously described; pregnant or lactating women: one tablet per day for 30 days). Approximately 50% of cases returned for the prophylactic dose after completing four days of treatment; anecdotal reports indicate that improvement in lip ulceration and fissuring among cases was seen in the majority of patients. Workers distributed vitamin B complex and micronutrient tablets to the rest of the population for prophylaxis at the community level (prophylactic dose administered as described above).

### Blood Specimens

In November 2009, under the guidance of UNICEF and in collaboration with the Uganda Virus Research Institute (UVRI), local lab personnel collected one to two tubes of whole blood from a convenience sample of 58 symptomatic cases (prior to treatment) and 18 asymptomatic individuals at three health facilities in two subcounties of Nakapiripirit District. Samples of frozen red blood cells washed in phosphate buffer solution were prepared and shipped per protocol to the University of Ulster nutrition laboratory for biochemical assessment of erythrocyte glutathione reductase activation coefficient (EGRac) to measure the presence of low concentrations of riboflavin (EGRac>1.4) [8].

Biochemical analysis confirmed riboflavin deficiency in 86.8% of all specimens with 84.5% of specimens from symptomatic individuals, and 94.4% of specimens from asymptomatic individuals. No statistically significant difference in deficiency status was observed by sex. The mean age of participants providing a blood specimen was 34.6 years (standard deviation, 15.9 years; range, five to 70 years).

### Ration Analysis

In January/February 2010, CDC personnel collected and analysed food ration planning and distribution records to determine the micro- and macro- nutrient content of food rations. Ration distribution data were only available for April to December 2009 in Kotido District, the district adjacent to Nakapiripirit. Thus Kotido calculations are presented as a proxy. While the Kotido calculations cannot provide an exact representation of the situation in Nakapiripit the planned ration for each district was the same, and similar distribution issues were faced in each district. Data were collected for the 18 distribution locations in Kotido and included the date of distribution, population size, and the metric tons of specific commodities. Nutval 2006 V2.2 was used to evaluate the energy, protein, fat, and micronutrient content of the general ratio [9], [10]. The calculated levels of micronutrients do not account for losses in storage (exposure to light) or cooking processes. Personnel based initial calculations upon commodity tonnage, population, and a planned 30 day cycle and then adjusted for the actual interval between distributions. While a ration may have been designed to last one month (30 days), personnel used the actual time between distributions to determine the daily nutritional content of the ration. Personnel also evaluated the ration for completeness of planned commodities in the ration package.

**Table 2** presents the commodities and quantities included in the planned ration for the Karamoja Region. Based on an assessment that the target population would be able to access 30% of their food and nutrient requirements on their own, the general food ration was set at a 70% ration or 1,470 kilocalories. The initial analysis of the ration for energy, fat, and protein showed a ration that was well balanced. While the level of micronutrients is well above the RDA for iodine (200%) and B_1_ (171%), the level of vitamin A and vitamin B_2_ provided by the ration meets 57% and 66%, respectively, of the RDA for the general population.

**Table 2:** Nutritional content of ration calculated on a planned 30 day cycle, Kotido District (neighboring Nakapiripirit District), Karamoja, Uganda, April – December 2009, NutVal 2006 V 2·2.

COMMODITY	RATION	ENERGY			PROTEIN		FAT	IRON		IODINE	VIT A	VIT B1			VIT B2	VIT B3		
	g/person/day	Kcal			g		g	mg		µg	µg RE	mg			mg	mg NE		
Maize grain, white	300	1,050			30.0		12.0	8.1		0	0	1.16			0.60	6.6		
Beans, dried	50	168			10.0		0.6	4.1		0	0	0.25			0.11	3.1		
Oil, vegetable^1^	15	133			0.0		15.0	0.0		0	135	0.00			0.00	0.0		
Corn soy blend^1^	30	120			5.4		1.8	3.8		0	150	0.13			0.21	3.0		
Salt, iodised^1^	5	0			0.0		0.0	0.0		300	0	0.00			0.00	0.0		
Ration Total	400	1,470			45.4		29.4	16.1		300	285	1.54			0.92	12.7		
Requirements	–	2,100			52.5		40.0	22.0		150	500	0.90			1·40	13.9		
% of requirements supplied by ration		70%			86%		74%	73%		200%	57%	171%			66%	92%		
% of energy supplied by protein or fat			–	12.4%		18.0%			–	–	–		–	–			–	

Note: NE = niacin equivalents, RE = retinol equivalents, VIT = vitamin.

1 WFP Specifications.

When adjusted for the actual time between distributions, a much different nutritional content was presented. Across the 18 distribution locations, only five of the nine planned distributions were conducted, with one location receiving only four distributions. The cycle (number of days between the distributions) varied greatly, ranging from 17 to 122 days, with an average length of 56·9 days (versus the planned 30 days). The average total energy across all distributions to the 18 locations was 1,032 kilocalories or 49% of the total energy requirement of 2,100 kilocalories and 70% of the planned ration of 1,470 kilocalories. Four percent of distributions between April and December 2009 achieved a 70% or higher energy content of the planned ration. Seventeen percent of distributions provided less than 30% of energy requirements. Regarding riboflavin, the range of mean riboflavin content was 0.23 to 1.6 mg per day (RDA = 1.4 mg), and 55% of distributions provided less than half of the RDA of riboflavin (**Table 3**).

**Table 3:** Number and mean duration of distributions and mean energy (Kcal) and riboflavin content of actual ration distributed across 18 locations in Kotido District, Karamoja, Uganda – April-December 2009.

Location	# of Distributions^a^	Mean Duration ofDistributions, days (Range)	Mean Kcal perDistribution (Range)	Mean Riboflavin Content,mg (Range)
A	5	49.7	1,133.9	0.85
		(28–74)	(596–1,575)	(0.62–1.6)
B	5	69.0	1,040.2	0.65
		(68–88)	(500–1,451)	(0.37–0.99)
C	5	41.0	1,276.5	0.80
		(40–95)	(464–1,451)	(0.29–0.99)
D	5	65.5	1,166.1	0.73
		(49–81)	(534–1,451)	(0.34–0.91)
E	5	55.0	1,222.7	0.76
		(42–68)	(645–1,451)	(0.4–0.91)
F	5	66.5	1,099.2	0.69
		(38–74)	(596–1,451)	(0.37–0.91)
G	5	53.0	1,141.5	0.72
		(46–68)	(649–1,451)	(0.41–0.91)
H	5	46.0	1,204.6	0.75
		(39–85)	(519–1,451)	(0.33–0.91)
I	5	63.0	1,175.6	0.74
		(37–77)	(573–1,451)	(0.36–0.91)
J	5	54.0	1,048.2	0.65
		(17–122)	(362–2,594)	(0.23–1.63)
K	5	65.0	1,157.1	0.73
		(39–79)	(558–1,451)	(0.35–0.91)
L	5	64.5	1,126.6	0.71
		(39–74)	(596–1,451)	(0.37–0.91)
M	5	54.0	1,485.9	1.1
		(29–79)	(558–1,521)	(0.35–0.95)
N	5	45.0	1,565.6	0.98
		(27–64)	(689–1,680)	(0.43–1.06)
O	5	65.0	1,450.7	0.91
		(46–79)	(558–1,451)	(0.35–0.91)
P	5	63.0	1,175.6	0.74
		(37–77)	(572–1,451)	(0.36–0.91)
Q	5	70.0	1,149.6	0.72
		(46–88)	(501–1,451)	(0.31–0.91)
R	4	64.0	1,157.6	0.73
		(36–77)	(573–1,451)	(0.36–0.91)

a Some locations increased the number of distribution sites per location; these were counted as one distribution as they covered the same population.

## Discussion

Findings in this investigation showed signs of AS in only 0.2% of the 179,172 screened individuals in Nakapiripirit District. Though documentation of other riboflavin deficiency investigations is limited, 0.2% is low in comparison to observations by Blanck et al., who observed signs of AS among 26.8% (95% CI: 22.3, 31.3) of a random sample of 463 adolescent Bhutanese refugees living in southeastern Nepal following the withdrawal of a vitamin-fortified cereal blend from the general ration in 1999 [6].

Evidence from these findings was insufficient to confirm an actual outbreak of riboflavin deficiency. However, this investigation provides documentation that attention towards micronutrient and calorie deficiencies is warranted among populations reliant on food assistance, as their access to micronutrient rich foods has been reduced by multiple factors. Data available were limited to data collected rapidly in response to an apparent outbreak of AS. Prior to this investigation, no data on baseline micronutrient status were available for this population. The lack of data lead to multiple challenges in responding to this suspected outbreak of riboflavin deficiency. Based on the experience of this investigation, the remainder of the article reviews challenges and limitations and provides recommendations for personnel working with nutritionally vulnerable populations receiving food assistance.

### Challenges and Limitations

While data on food security, anthropometric z-scores, and feeding program admissions among this population were plentiful, summarizing trends in nutritional status was not possible due to methodological differences and seasonal inconsistencies in available data. Furthermore, available data lacked information on micronutrient status to help characterize the extent of the suspected outbreak.

Though findings from the rapid assessment in Loregedwat Subcounty prompted a wider mass screening throughout Nakapiripirit District, fewer AS cases were identified during the mass screening than expected. This may be due to differences in 1) the screening locations (health center versus general population), 2) the distribution of cases within Lorengedwat Subcounty versus the rest of Nakapiripirit District, 3) screening methodology applied (the rapid assessment was implemented very quickly, while the mass screening was conducted after an organized training), or 4) timing of the mass screening, which followed a mass distribution of vitamin B complex and micronutrient tablets in Lorengedwat Subcounty. The lower than expected coverage for mass screening activities was likely a result of population out-migration due to drought and crop failure. Out-migration impeded follow-up activities and compliance with prophylaxis. VHTs were encouraged to follow-up with those who did not return for the prophylactic dose and bring them to the health center. Distributing treatment and/or the follow-up prophylactic course at the community level, perhaps through volunteer health workers that remain with the community despite migration, may reduce drop-out and facilitate follow-up.

Challenging technical and logistical requirements of biologic testing limited the generalizability of laboratory data. While biologic testing confirmed the presence of riboflavin deficiency in a clinic population, testing for multiple micronutrients from a larger, representative sample of individuals would have provided more conclusive documentation of the micronutrient status of the population. Furthermore, the observation that riboflavin deficiency was higher in asymptomatic than symptomatic individuals may be an indication of broader underlying deficiency among this population; however, sampling limitations restrict the conclusiveness of these findings,

### Recommendations

Experiences responding to this suspected outbreak yielded the following lessons learned and recommendations for personnel working with nutritionally vulnerable populations receiving food assistance:

#### Ongoing nutrition monitoring and surveillance

To fully document a population’s micronutrient status and evaluate the impact of interventions to reduce deficiencies, monitoring and surveillance measures are necessary. Nutrition assessments are common among food assistance beneficiaries and can be used as a basis to which micronutrient assessment can be integrated, thus reducing the associated cost burden. Assessments should be conducted systematically and the timing of implemented surveys coordinated to allow for trend analysis, which is critical to assess nutritional status. Implementing surveys at a regular time interval, which can elucidate seasonal patterns of deficiencies, will aid with planning.

#### Enhanced monitoring and surveillance during an outbreak of suspected micronutrient deficiency

If an outbreak of a suspected micronutrient deficiency has been detected, affected individuals should be treated and monitored through a follow-up visit to track change of symptoms to monitor persistence of symptoms and appropriateness of treatment. During the investigation of a potential micronutrient deficiency outbreak, it is important to recognize that micronutrient deficiencies do not happen in isolation; where one deficiency is observed, deficiencies among other micronutrients are probably prevalent [11], [12]. Where feasible, biochemical testing for a panel of micronutrients should be considered among a representative sample of the population at risk. Biochemical testing is accompanied by many challenges, including expense, the need for qualified staff and sophisticated laboratory equipment for analysis, and difficulties maintaining a proper cold chain in field settings. Provisions for accommodating such testing should be discussed in advance. Lastly, nutrition surveys conducted among the population should adhere to methodologically sound practices in order to ensure generalizability of survey results; a thorough investigation among a small but representative population will likely yield more useful results than a non-representative rapid survey of the population.

#### Training and sensitization about micronutrient deficiencies among health personnel

Increased sensitivity to indications of micronutrient deficiencies will improve treatment of individuals suffering from a deficiency, as well as expedite identification of potential outbreaks. Practical treatment protocols tailored to the specific micronutrient profile of the affected population should be readily accessible by clinic staff. As mentioned above, it is crucial that affected individuals be followed to track adherence, compliance, and treatment outcomes. Active efforts must be made to facilitate follow-up contact with affected individuals, which can be achieved through a return clinic visit by the patient, mobile phone technology, and/or by a home visit by a community health worker.

#### Sensitization of the population about locally-available food

Populations receiving food assistance should be sensitized to meet the balance of their daily food requirements (in the case of Karamoja, 30%) with nutritious-appropriate food found locally. In the context of Karamoja, the following ribovlavin-rich foods are available locally and could be encouraged among populations: sesame seeds (*simsim*), liver, dried herbs, and chilli. While these foods are available, the accessibility of the micronutrient rich foods must be assessed to ensure that households can access these items.

#### Identify ways to improve micronutrient interventions for populations receiving food assistance

The micronutrient profile of populations who will receive food assistance should be studied to identify better-tailored nutritional interventions. Where children (six to 59 months) are at risk for micronutrient deficiency, multi-micronutrient powders (MNPs, e.g. Sprinkles) are a potential intervention [13]. Appropriate micronutrient interventions should be based upon the context of the geographic area, disease profile, and existing micronutrient intake of the population. If powders are to be used, to the extent that is feasible, formative research is recommended to identify appropriate marketing and distribution mechanisms. Also, a strong educational campaign should be implemented to ensure correct usage, and a monitoring component to the program should be implemented to assess compliance and utilization among the targeted population [13].

Integration of CSB+ (corn soy blend) as a commodity in the food ration can also be considered to improve the overall micronutrient profile of a population. CSB has been reformulated to CSB+ to improve the micronutrient profile of rations by increasing the bioavailability of the contained micronutrients and the inclusion of type II nutrients that are the basic building blocks required for sustaining tissue (including nitrogen, potassium, sodium, zinc, and essential amino acids); as the body cannot store type II nutrients, deficiency of these nutrients results in growth failure or weight loss. Inclusion of fortified blended foods in general rations has provided prevention against micronutrient deficiencies in some emergency settings [14]. The feasibility of distributing new formulations of CSB with enhanced micronutrient profiles in the general ration in settings where populations are at risk for micronutrient deficiencies should be explored, and policies supporting the procurement and distribution of such formulations should be implemented.

This investigation documents that micronutrient deficiencies persist among populations in emergency settings, placing them at further risk of morbidity. Ongoing nutrition monitoring and surveillance should be conducted regularly among populations receiving food assistance, and health personnel working with such populations should be sensitized and trained on micronutrient deficiencies. When a suspected micronutrient deficiency outbreak is detected, monitoring and surveillance should be enhanced. Preventing outbreaks of micronutrient deficiency through dietary measures should be continued through advocacy to consume locally-available, nutritious foods and to improve the adequacy of food rations. .
